# Nanostructured Polyphase Catalysts Based on the Solid Component of Welding Aerosol for Ozone Decomposition

**DOI:** 10.1186/s11671-015-1186-7

**Published:** 2015-12-09

**Authors:** Tatyana Rakitskaya, Alla Truba, Alim Ennan, Vitaliya Volkova

**Affiliations:** Odessa I.I. Mechnikov National University, 2 Dvoryanskaya str., Odessa, 65082 Ukraine; Physicochemical Institute of Environment and Human Protection, 3, Preobrazhenskaya St., Odessa, 65082 Ukraine

**Keywords:** Welding aerosol, Solid component, Characterization, Ozone decomposition, Air purification

## Abstract

Samples of the solid component of welding aerosols (SCWAs) were obtained as a result of steel welding by ANO-4, TsL‑11, and UONI13/55 electrodes of Ukrainian manufacture. The phase compositions of the samples, both freshly prepared (FP) and modified (M) by water treatment at 60 °C, were studied by X-ray phase analysis and IR spectroscopy. All samples contain magnetite demonstrating its reflex at 2*θ* ~ 35° characteristic of cubic spinel as well as manganochromite and iron oxides. FP SCWA-TsL and FP SCWA-UONI contain such phases as СaF_2_, water-soluble fluorides, chromates, and carbonates of alkali metals. After modification of the SCWA samples, water-soluble phases in their composition are undetectable. The size of magnetite nanoparticles varies from 15 to 68 nm depending on the chemical composition of electrodes under study. IR spectral investigations confirm the polyphase composition of the SCWAs. As to IR spectra, the biggest differences are apparent in the regions of deformation vibrations of M–O–H bonds and stretching vibrations of M–O bonds (M–Fe, Cr). The catalytic activity of the SCWAs in the reaction of ozone decomposition decreases in the order SCWA-ANO > SCWA-UONI > SCWA-TsL corresponding to the decrease in the content of catalytically active phases in their compositions.

## Background

Welding aerosols consisting of solid and gaseous components are evolved as a result of complex physical and chemical processes taking place in welding. The solid component of welding aerosol (SCWA) is the air-dispersed particulate formed out of the welding arc in consequence of the oxidation and condensation of vapors of components of electrode coatings, welding fluxes, and metals. The gaseous component of welding aerosol (GCWA) is a result of complex reactions leading to the formation of HF, SiF_4_, NO_X_, and O_3_ [1, 2]. Ozone is one of the most hazardous among the listed gases: its maximum permissible concentration is 0.1 mg/m^3^. Taking into consideration volumes and technologies of welding and related industries as well as materials used for them, it is important not only to decrease the toxic effect of SCWAs and GCWAs on the environment and operating personnel but also to recycle solid wastes in order to obtain industrial products.

As appears from literature, chemical and phase compositions of SCWAs depend on a nature of weld metals and electrodes, materials of electrode coatings, and welding conditions [2–12]. Our investigations show that magnetite and manganochromite contained in compositions of SCWAs formed in the process of steel welding by some electrodes manufactured in Ukraine exhibit stable catalytic behavior in the reaction of ozone decomposition for the wide range of ozone concentrations (1–100 mg/m^3^) [13, 14]. It can be expected that an increase in concentrations of catalytically active phases in SCWAs obtained as a result of removing impurity phases and phases inactive in the reaction of ozone decomposition would lead to a change in kinetic and stoichiometric parameters of the reaction.

As follows from literary data [15–18], nanoparticles of magnetite (FeFе_2_O_4_) and its metal-substituted forms (МFе_2_O_4_) are prepared by their precipitation in an aqueous medium at temperatures not exceeding 60 °C. Such a treatment of SCWAs with water at the mentioned temperature can be used also as a mild technique not leading to structural changes in magnetite and its metal-substituted forms.

The aim of the work is to compare the phase compositions of freshly prepared and modified samples of the SCWAs obtained as a result of steel welding with the help of electrodes differing by their chemical compositions and to determine the catalytic activity of the SCWAs in the reaction of ozone decomposition.

## Materials and Methods

The welding fume fractions of the SCWAs with an aerodynamic diameter of ≤1 μm formed in the process of metal arc welding by 3-mm-diameter electrodes with either rutile (ANO-4 (ISO 2560 E432R 21)) or carbonate-fluorite (TsL-11 (ISO E19.9NbB20) and UONI 13/55 (ISO 2560 E434B20)) electrode coatings were selected for the study. Welding was performed under conditions of positive current, reverse polarity, *U* = 33 V, *I* = 140–150 A, and the welding speed of 4.5 mm/s [2, 3]. Elemental analysis of the two SCWAs showed that both predominantly consisted of iron and manganese, and SCWA-TsL-11 was characterized by a much higher content of chromium and nickel [3].

In order to prepare modified samples, 20 mL of distilled water was added to 1 g of a SCWA sample and the suspension obtained was kept at 60 °C for 2 h under continuous stirring. After that, the particulate was filtered and dried at 110 °C till constant weight.

SCWA samples were characterized by X-ray diffraction phase analysis and IR spectroscopy. In addition, they were tested in the reaction of low-temperature ozone decomposition.

The samples were identified based on X-ray diffraction phase analysis data recorded on a Siemens D500 diffractometer (CuK_α_ radiation, *λ* = 1.54178 Å) with a secondary beam graphite monochromator. The phases were identified with the help of International Centre for Diffraction Data (ICDD) PDF-1 databases provided as a part of the Siemens D500 diffractometer software.

Infrared analysis was carried out using a Perkin Elmer FT-IR spectrometer with a resolution of 4 cm^−1^; pellets consisting of 1 mg of the material under study and 200 mg of KBr were compressed under pressure of 7 tons/cm^2^ for 30 s.

The catalyst samples (0.5 g) were tested using a gas-flow setup with a fixed bed reactor at 20 °C, relative humidity of 65 %, and the linear velocity (*u*) of an ozone-air mixture (OAM) equal to 3.2 cm/s. The ozone decomposition was monitored by measuring the final ozone concentration (${C}_{{\mathrm{O}}_3}^{\mathrm{f}}$). The initial ozone concentration (${C}_{{\mathrm{O}}_3}^{\mathrm{in}}=100\ \mathrm{mg}/{\mathrm{m}}^3$) and ${C}_{{\mathrm{O}}_3}^{\mathrm{f}}$ were measured by a Tsyclon-Reverse optical analyzer with a detection limit of 1 mg/m^3^. The reaction rate (*W*) calculations based on the data of ozone concentration changing after OAM passing through the static bed of the catalyst were made using the following equation:

1 $$ W=\frac{\omega \left({C}_{{\mathrm{O}}_3}^{\mathrm{in}} - {C}_{{\mathrm{O}}_3}^{\mathrm{f}}\right)}{m_{\mathrm{s}}},\mathrm{mol}/\left(\mathrm{g}\times \mathrm{s}\right), $$

where *ω* = 1.67 × 10^−2^ is the OAM volume flow rate, L/s; ${C}_{{\mathrm{O}}_3}^{\mathrm{in}}$ and ${C}_{{\mathrm{O}}_3}^{\mathrm{f}}$ are the initial and final ozone concentrations in the OAM, respectively, mol/L; and *m*_s_ is the mass of the catalyst sample, g.

The reaction rate values measured after 1 min of OAM passing named as the initial reaction rate, *W*_in_, were used to characterize the process.

The first-order reaction rate constant with reference to ozone, *k*_1_, was graphically calculated using the following equation:

2 $$ {k}_1=\frac{1}{\tau } ln\frac{C_{{\mathrm{O}}_3}^{\mathrm{in}}}{C_{{\mathrm{O}}_3}^{\mathrm{f}}}, {\mathrm{s}}^{{}^{-}1}, $$

where *τ* is reaction time, s.

The reaction rate constant, *k*_1/2_, was quantified for the half-conversion time, *τ*_1/2_, i.e., for the moment of time when the degree of ozone decomposition became equal to 50 %, as follows:

3 $$ {k}_{1/2}=\frac{0.69}{\tau_{1/2}},\ {\mathrm{s}}^{{}^{-}1}. $$

The amount of ozone entered into the reaction up to the moment of experiment termination (*Q*_exp_, moles of О_3_) was calculated as the square of the corresponding ozonogram plotted as Δ*C*_O3_ vs. *τ* function.

## Discussion

### XRD Characterization

**Fig. 1:**
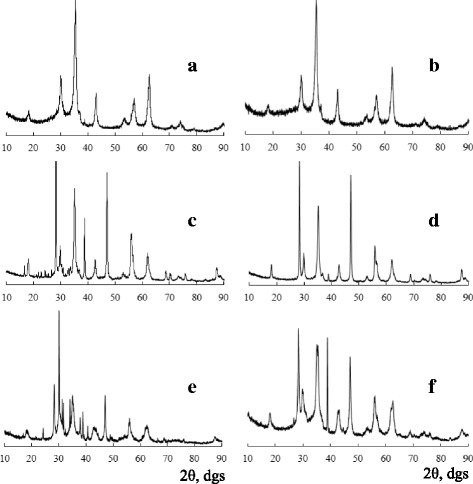
X-ray diffraction patterns of freshly prepared (**a**, **c**, **e**) and modified (**b**, **d**, **f**) SCWAs: **a**, **b** ANO; **c**, **d** TsL; **e**, **f** UONI

X-ray diffraction patterns of freshly prepared (Fig. 1a, c, e) and modified (Fig. 1b, d, f) SCWA samples show a substantial difference in positions, intensities, and numbers of reflections; however, all of them are characterized by a high degree of crystallinity.

Tables 1, 2, and 3 show results of analysis of our data related to the phase composition of samples under study and their X-ray spectral parameters: angles of reflection, 2*θ*; interplanar spacings, *d* (Ǻ), both experimental and reference; and normalized intensities, *I*_N_. Taking into account the chemical composition of both electrode wires and electrode coatings, identifying phases in SCWA compositions, we considered the possibility of a formation of various spinels, intermetallic compounds, metal oxides, fluorides, silicate forms, carbonates, etc. Since superposition of reflections occurs in many cases, we must pay first-priority attention to the presence of individual reflections of each phase in the X-ray spectra. As can be seen from Tables 1, 2, and 3, the SCWA samples are polyphase. Seven crystalline phases, i.e., magnetite (Fe_3_O_4_) [ICPDS 19-0629], manganochromite ((Mn,Fe)(Cr,V)_2_O_4_) [ICPDS 31-0630], manganese oxide (Mn_3_O_4_) [ICPDS 13-0162], carbonates of potassium (K_2_CO_3_) and sodium (Na_2_CO_3_) [19], potassium chromate (K_2_CrO_4_), and dichromate K_2_Cr_2_O_7_ [19], have been identified in freshly prepared (FP) SCWA-ANO (Table 1). Phase compositions of FP SCWA-TsL (Table 2) and FP SCWA-UONI (Table 3) are more complicated. In addition to the phases found in the first sample (Table 1), iron oxides (β-Fe_2_O_3_⋅H_2_O and Fe_2_O_3_) [19], calcium fluoride (CaF_2_) [ICPDS 35-0816], and magnesium silicate (MgSiO_3_) [ICPDS 11-0273] have been identified. The most intense reflections were observed for phase mixtures.

**Table 1:** X-ray spectral parameters and the phase composition of SCWA-ANO

Phase Ref.	Freshly prepared sample				Modified sample			
	2*θ*, °	*I*_N_	*d* (Ǻ)		2*θ*, °	*I*_N_	*d* (Ǻ)	
			Exp.	Ref.			Exp.	Ref.
(Mn,Fe)(Cr,V)_2_O_4_	18.212	85	4.867	4.85	18.362	219	4.828	4.85
	36.654	70	2.449	2.44	–	–	–	–
	–	–	–	–	42.784	210	2.112	2.12
	56.402	121	1.630	1.63	56.453	197	1.629	1.63
	61.727	149	1.502	1.50	–	–	–	–
	–	–	–	–	62.402	369	1.487	1.50
Fe_3_O_4_	35.389	1000	2.534	2.55	35.546	999	2.524	2.55
	36.978	87	2.429	2.42	37.204	222	2.415	2.42
	43.027	297	2.100	2.10	43.186	344	2.093	2.10
	53.414	58	1.714	1.71	–	–	–	–
	57.001	219	1.614	1.61	57.015	295	1.614	1.61
	62.587	463	1.483	1.483	62.724	528	1.480	1.483
	73.957	75	1.281	1.279	74.154	121	1.278	1.279
	79.004	22	1.211	1.210	78.943	70	1.212	1.210
Mn_3_O_4_	53.638	57	1.707	1.70	53.712	147	1.705	1.70
K_2_CO_3_	34.189	95	2.620	2.61	Not detected			
Na_2_CO_3_	56.058	90	1.639	1.62	Not detected			
	62.071	250	1.494	1.48	Not detected			
	63.278	62	1.470	1.48	Not detected			
K_2_CrO_4_	38.826	38	2.317	2.32	Not detected			
K_2_Cr_2_O_7_	30.809	75	2.900	2.85	Not detected			
	53.091	48	1.724	1.73	Not detected			
(Mn,Fe)(Cr,V)_2_O_4_;	30.033	328	2.973	2.99	30.159	439	2.961	2.99
Fe_3_O_4_;				2.97				2.97
K_2_CrO_4_				2.96				2.96

**Table 2:** X-ray spectral parameters and the phase composition of SCWA-TsL

Phase	Freshly prepared sample				Modified sample			
	2*θ*, °	*I*_N_	*d* (Ǻ)		2*θ*, °	*I*_N_	*d* (Ǻ)	
			Exp.	Ref.			Exp.	Ref.
(Mn,Fe)(Cr,V)_2_O_4_	18.072	122	4.905	4.89	18.179	102	4.876	4.89
	36.685	50	2.448	2.44	36.766	52	2.442	2.44
Fe_3_O_4_	35.095	658	2.555	2.55	35.201	567	2.548	2.55
	43.104	48	2.097	2.10	43.113	59	2.097	2.10
	73.622	24	1.286	1.279	73.675	30	1.285	1.279
β-Fe_2_O_3_⋅Н_2_О	46.383	37	1.956	1.96	46.077	16	1.968	1.96
	68.297	16	1.372	1.38	68.798	71	1.363	1.38
Mn_3_O_4_	73.876	21	1.282	1.28	73.315	33	1.290	1.28
	78.794	11	1.214	1.21	78.534	13	1.217	1.21
	89.748	11	1.092	1.09	89.418	17	1.095	1.09
CaF_2_	46.999	871	1.932	1.93	47.130	881	1.927	1.93
	68.647	76	1.365	1.365	68.798	271	1.363	1.365
	87.378	113	1.115	1.115	87.531	118	1.114	1.115
MgSiO_3_	30.804	50	2.900	2.908	30.628	22	2.917	2.900
	61.330	49	1.510	1.499	61.513	60	1.506	1.499
K_2_CO_3_	31.670	11	2.822	2.80	Not detected			
K_2_CrO_4_	29.249	47	3.051	3.07	Not detected			
	30.310	83	2.946	2.96	Not detected			
CaF_2_; MgSiO_3_	28.251	1000	3.156	3.154	28.386	999	3.142	3.154
				3.170				3.170
K_2_CO_3_	34.486	117	2.599	2.61	Not detected			
Na_2_CO_3_				2.60				
K_2_CrO_4_; NaF; β-Fe_2_O_3_⋅Н_2_О	38.802	455	2.319	2.321	38.910	49	2.313	2.29
				2.32				2.32
				2.29				2.29
(Mn,Fe)(Cr,V)_2_O_4_; β-Fe_2_O_3_⋅Н_2_О; Fe_3_O_4_; KF⋅2H_2_O	42.631	147	2.119	2.12	42.747	123	2.114	2.12
				2.10				2.10
				2.10				2.10
				2.12				2.12
(Mn,Fe)(Cr,V)_2_O_4_; β-Fe_2_O_3_⋅Н_2_О; KF⋅2H_2_O	61.969	200	1.496	1.50	62.051	173	1.495	1.50
				1.494				1.494
				1.495				1.495
Fe_3_O_4_ ; β-Fe_2_O_3_⋅Н_2_О; Fe_3_O_4_; Na_2_CO_3_	62.361	97	1.487	1.483	62.471	88	1.485	1.483
				1.485				1.485
				1.486				1.486
				1.482				1.482

**Table 3:** X-ray spectral parameters and the phase composition of SCWA-UONI

Phase	Freshly prepared sample				Modified sample			
	2*θ*, °	*I*_N_	*d*, Å		2*θ*, °	*I*_N_	*d*, Å	
			Exp.	Ref.			Exp.	Ref.
(Mn,Fe)(Cr,V)_2_O_4_	18.055	54	4.909	4.89	18.068	144	4.906	7.89
	29.740	131	3.001	2.99	29.797	299	2.996	2.99
	–	–	–	–	42.425	168	2.129	2.12
	56.066	160	1.639	1.63	56.130	310	1.637	1.639
	61.823	95	1.499	1.50	61.806	257	1.50	1.50
Fe_3_O_4_ (magnetite)	35.465	177	2.529	2.53	35.418	726	2.532	2.53
	42.880	89	2.107	2.10	42.899	206	2.106	2.10
	–	–	–	–	56.837	134	1.619	1.61
	62.104	111	1.486	1.48	62.403	316	1.487	1.485
Fe_2_O_3_ (goethite, hematite)	24.103	44	3.689	3.68	–	–	–	–
	35.844	87	2.503	2.51	35.961	174	2.495	2.51
	62.134	106	1.493	1.485	62.137	266	1.493	1.485
β-Fe_2_O_3_⋅Н_2_О (agacansite)	39.243	31	2.293	2.29	39.502	25	2.280	2.29
	43.055	92	2.099	2.10	43.112	213	2.097	2.10
Mn_3_O_4_	28.749	66	3.103	3.08	–	–	–	–
	42.664	88	2.117	2.10	42.634	215	2.119	2.10
	62.689	103	1.481	1.48	62.557	339	1.484	1.48
CaF_2_	28.282	357	3.153	3.15	28.319	1000	3.149	3.15
	47.023	317	1.931	1.93	47.075	751	1.929	1.93
	68.663	31	1.366	1.365	68.546	54	1.368	1.365
	87.361	55	1.115	1.115	87.303	69	1.116	1.115
K_2_CO_3_	31.768	208	2.815	2.80	Not detected			
	37.831	141	2.376	2.37	Not detected			
Na_2_CO_3_⋅Н_2_О	40.628	95	2.220	2.24	Not detected			
K_2_CrO_4_	29.176	40	3.058	3.07	Not detected			
	53.028	23	1.726	1.72	Not detected			
	57.110	39	1.612	1.61	Not detected			
MgSiO_3_	30.844	109	2.897	2.908	30.672	94	2.912	2.908
	54.978	27	1.669	1.66	55.266	57	1.660	1.66
	68.914	16	1.361	1.359	68.852	68	1.362	1.359
Fe_3_O_4_; Mn_3_O_4_; K_2_CrO_4_; Na_2_CO_3_	30.073	1000	2.969	2.97	30.046	268	2.972	2.97
				2.98				2.98
				2.96				2.96
				2.96				2.96
K_2_Cr_2_O_7_; Mn_3_O_4_	31.224	239	2.862	2.87	31.090	64	2.874	2.87
				2.87				2.87
K_2_CO_3_; β-Fe_2_O_3_⋅Н_2_О	34.090	255	2.628	2.61	33.914	58	2.671	2.61
				2.64				2.64
(Mn,Fe)(Cr,V)_2_O_4_; β-Fe_2_O_3_⋅Н_2_О; MgSiO_3_	35.050	279	2.558	2.55	35.031	775	2.559	2.55
				2.55				2.55
				2.551				2.551
β-Fe_2_O_3_⋅Н_2_О; Mn_3_O_4_; K_2_CrO_4_; NaF	38.836	184	2.317	2.29	38.854	800	2.316	2.29
				2.36				2.36
				2.32				2.32
				2.32				2.32
β-Fe_2_O_3_⋅Н_2_О; CaF_2_; MgSiO_3_	55.760	130	1.647	1.648	55.941	340	1.642	1.648
				1.647				1.647
				1.642				1.642

For modified (M) SCWAs, the analysis of X-ray diffraction patterns (Fig. 1b, d, f) and the information presented in Tables 1, 2, and 3 show a decrease in the number of reflections and a change in their intensity ratios due to the loss of water-soluble phases. M SCWAs contain phases catalytically active in redox reactions (CAP) such as magnetite, manganochromite, iron oxides, and their mixtures. Taking into account integral intensities of the corresponding reflections, the content (%) of CAPs could be estimated for each SCWA sample (Table 4). It decreases in the order SCWA-ANO > SCWA-UONI > SCWA-TsL. Judging from the peak at 2*θ* ~ 35^о^ (311), all SCWA samples contain magnetite in the form of ferrites with a cubic spinel structure [10, 20–27]. Based on this fact, we have estimated the unit cell parameter (*a*). The values of *a* parameter obtained by us (Table 4) are in agreement with literature ones for the cubic unite cell parameter of FeFe_2_O_4_, i.e., 8.380 Ǻ [23], 8.199 Ǻ [24], and 8.394 Ǻ [25]. Slight differences in the literature values can be caused by differences in techniques used for ferrite preparation. For metal-substituted ferrites, (Zn,Mn)Fe_2_O_4_, *a* is in the range from 8.459 to 8.472 Ǻ [28].

**Table 4:** CAP contents, unit cell parameters, and sizes of magnetite nanoparticles estimated from (311) reflection of Fe_3_O_4_

Sample	Content of CAP, %	*а*, Ǻ	*D*, nm
FP SCWA-ANO	81	8.404	18.0
M SCWA-ANO	97	8.371	15.0
FP SCWA-TsL	51	8.474	23.0
M SCWA-TsL	52	8.451	20.5
FP SCWA-UONI	54	8.388	68.0
M SCWA-UONI	78	8.398	31.5

Using the well-known Scherrer equation, the sizes of magnetite nanoparticles (*D*, nm) were estimated based on the breadth at half-peak height of the X-ray diffraction (XRD) line corresponding to (311) reflections for the freshly prepared and modified SCWA samples (Table 4). It can be seen that the sizes of magnetite nanoparticles depend, other things being equal, on the chemical composition of electrodes and the highest *D* values are for the SCWA-UONI samples. It should be noted that, despite the significant differences in *D* values presented in Table 4, it does not contradict with the data spread reported in literature [10, 25–27, 29, 30]. Depending on techniques and conditions of magnetite preparation used in those works, *D* values vary from 11 to 52 nm.

### IR Characterization

**Fig. 2:**
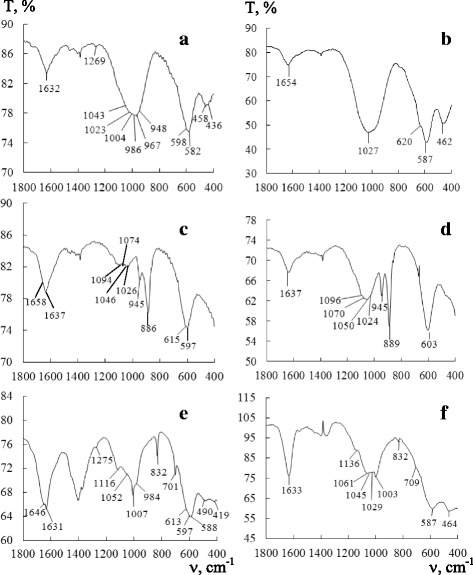
IR spectra of the freshly prepared (**a**, **c**, **e**) and modified (**b**, **d**, **f**) SCWAs: **a**, **b** ANO; **c**, **d** TsL; **e**, **f** UONI

The difficulties in differentiation of mixed and individual characteristic vibrations of M–O and M–OH bonds (M—metal) make IR spectral investigations of polyphase systems, specifically SCWAs, very complicated. The data obtained in our earlier work [14] for freshly prepared SCWA-ANO and SCWA-TsL show that the biggest differences are observed in the region of 1700–400 cm^−1^. Therefore, Fig. 2 shows only that spectral region for the freshly prepared (panels a, c, e) and modified (panels b, d, f) SCWA samples. As can be seen, the bands characterizing deformation vibrations of water molecules in the freshly prepared samples are not simple that indicates energy inhomogeneity of surface sites occupied by water molecules.

For identification of characteristic vibration frequencies of M–O and M–OH bonds in the IR spectra, literary data for both metal oxides [28, 31–33] and spinels [21–24, 34, 35] whose compositions are similar to those determined in the SCWAs by X-ray phase analysis were used. IR spectra of the freshly prepared SCWA samples (Fig. 2a, c, e) contain many absorption bands of different intensity and resolution that confirms the structural-phase inhomogeneity of the samples. An intense complex-shaped band is detected in the spectral region of 1250–850 cm^−1^ for FP SCWA-ANO. Its components at 1043 and 1023 cm^−1^ can be assigned to deformation vibrations of Fe–O–H bonds in spinel whereas absorption bands at 1004, 986, 967, and 948 cm^−1^ can be attributed to stretching vibrations of Cr–O bonds in the case of a coordinatively unsaturated chromium atom. A low-intensity band at 1269 cm^−1^ is assigned to vibrations of a Fe–O–H bond in spinel. Intense bands at 598 and 582 cm^−1^ as well as moderate-intensity bands at 458 and 436 cm^−1^ are caused by vibrations of Fe–O bonds in the case of iron cations located in tetrahedral and octahedral positions of a spinel structure. For FP SCWA-TsL, deformation vibrations of a Fe–O–H bond in spinel are detected at 1094, 1074, 1046, and 1026 cm^−1^ as very weak unassisted bands separated from one another by intervals of 20 cm^−1^. A similar series of bands was observed in the spectrum of (Zn,Mn)Fe_2_O_4_ spinel [28]. For the FP SCWA-TsL sample, a distinctive feature of its spectrum is a clear-cut resolution of the bands at 945 and 886 cm^−1^ assigned to stretching vibrations of Cr–O when the chromium atom is coordinatively saturated [32]. An intense band centered at 597 cm^−1^ with a shoulder at 615 cm^−1^ is a combined one and corresponds to vibrations of Fe–O bonds in both a spinel structure and free iron(III) oxides. Deformation vibrations of a Fe–O–H bond in a spinel structure of FP SCWA-UONI are detected as high-resolution low-intensity bands at 1275 and 1116 cm^−1^ with a shoulder at 1052 cm^−1^. Compared with the spectra of other freshly prepared SCWAs, the spectrum of FP SCWA-UONI has some differences in the region of Cr–O stretching vibrations. They appear in the composition of a combined band having its maximum at 1007 and a shoulder at 984 cm^−1^ and also in the appearance of a sharp band at 832 cm^−1^. Some absorption bands detected in the region of 800–400 cm^−1^ are assigned to stretching vibrations of Fe–O–H bonds: 597 and 490 cm^−1^—in spinels—and 701, 613, 588, and 419 cm^−1^—in individual iron(III) oxides.

Figure 2 shows IR spectral fragments covering the region of 1800–400 cm^−1^ for M SCWA-ANO (panel b), M SCWA-TsL (panel d), and M SCWA-UONI (panel f). It can be seen that the modification results in some structural-phase changes. In the spectral region corresponding to deformation vibrations of water molecules, only one band is detected for each sample: 1634 cm^−1^ for M SCWA-ANO, 1637 cm^−1^ for M SCWA-TsL, and 1633 cm^−1^ for M SCWA-UONI. As expected, the biggest changes take place in the region of 1250–850 cm^−1^ which belongs to deformation vibrations of M–O–H bonds most sensitive to changes in spinel structures [28]. For example, for M SCWA-ANO, a moderate-intensity band appeared at 1027 cm^−1^ (Fe–OH) instead of the intense complex-shaped band detected in the spectral region of 1250–850 cm^−1^ for FP SCWA-ANO. Absorption bands assigned to Cr–O stretching vibrations do not appear independently in the M SCWA-ANO spectrum because of a low chromium content. Most likely, these bands are superposed with a more intense band characteristic for a Fe–OH bond in spinel. In the M SCWA-TsL spectrum, a broad band centered at 1050 cm^−1^ with shoulders at 1096, 1070, and 1024 cm^−1^ can be attributed to stretching vibrations of a Fe–OH bond in spinel, and individual bands at 945 and 889 cm^−1^ do not disappear nor shift due to a high chromium content in the electrode. Substantial changes are observed in the spectral region of 1250–850 cm^−1^ for M SCWA-UONI. A combined band located there broadens, and 1136, 1061, 1045, and 1029 cm^−1^ frequencies in its high-frequency component are assigned to Fe–O–H vibrations in spinel whereas a 1003 cm^−1^ frequency located in its low-frequency component is attributed to the vibration of a Cr–O bond. The intensity of a band at 832 cm^−1^ markedly decreases. Two bands, the first one centered at 587 cm^−1^ with a shoulder at 709 cm^−1^ and the second one located at 464 cm^−1^, are caused by vibrations of Fe–O bonds with iron cations disposed in tetrahedral and octahedral positions in a spinel structure.

### Testing SCWA Samples in the Reaction of Ozone Decomposition

**Fig. 3:**
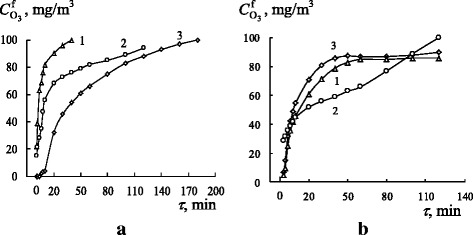
Time dependences of the final ozone concentration, ${C}_{{\mathrm{O}}_3}^{\mathrm{f}}$, in the course of ozone decomposition by freshly prepared (**a**) and modified (**b**) SCWAs. *1* SCWA-TsL, *2* SCWA-UONI, and *3* SCWA-ANO (${C}_{{\mathrm{O}}_3}^{\mathrm{in}} = 100\ \mathrm{mg}/{\mathrm{m}}^3,{m}_{\mathrm{s}} = 0.5\kern0.5em \mathrm{g}$)

Figure 3 shows time dependences of the final ozone concentration, ${C}_{{\mathrm{O}}_3}^{\mathrm{f}}$, in the course of ozone decomposition by the freshly prepared (panel a) and modified (panel b) SCWA samples.

As can be seen, the duration of the reaction and profiles of kinetic curves depend on phase compositions of the samples and the longest reaction time is observed for FP SCWA-ANO. Initial portions of the kinetic curves are most sensitive to alterations in SCWA compositions. During the first 10 min, ${C}_{{\mathrm{O}}_3}^{\mathrm{f}}$ values increase only from 1 to 5 mg/m^3^ for FP SCWA-ANO, from 15 to 58 mg/m^3^ for FP SCWA-TsL, and from 20 to 80 mg/m^3^ for FP SCWA-UONI.

Modification of SCWA samples causes significant changes in the kinetics of ozone decomposition. M SCWA-ANO and M SCWA-TsL decompose ozone with attainment of a steady-state mode approximately in 60 min after passing the OAM through the catalyst bed. A curve 2 corresponding to M SCWA-UONI is located lower than the curves for the first two samples; however, no steady-state mode is observed for it. The kinetic parameters (*W*_in_, *k*_1_, and *k*_1/2_) for the reaction of ozone decomposition by the SCWAs under study and amounts of ozone entered into the reaction (*Q*_exp_) are summarized in Table 5.

**Table 5:** Kinetic parameters for the reaction of ozone decomposition by freshly prepared and modified SCWAs (${C}_{{\mathrm{O}}_3}^{\mathrm{in}} = 100\ \mathrm{mg}/{\mathrm{m}}^3,{m}_{\mathrm{s}} = 0.5\ \mathrm{g}$)

Sample	*W*_in_ × 10^8^, mol/L × s	*k*_1_ × 10^3^, s^−1^	*k*_1/2_ × 10^4^, s^−1^	*Q*_exp_ × 10^5^, О_3_ moles
FP SCWA-ANO	7.00	4.4	3.3	8.3
M SCWA-ANO	6.51	3.7	14.4	4.0
FP SCWA-TsL	5.76	2.2	24.0	0.9
M SCWA-TsL	6.65	3.8	8.8	4.9
FP SCWA-UONI	5.95	2.4	13.8	4.4
M SCWA-UONI	4.97	0.8	6.1	6.0

Values of *k*_1_ (calculated for the initial portions of the kinetic curves) and *k*_1/2_ (calculated at the time of half-conversion of ozone) are not equal being evidence of ozone decomposition proceeding by the radical chain mechanism (much the same as in the case of complex compounds [36, 37], metal oxides [38], etc.). Magnetite, manganochromite, and iron(III) oxides can be considered as catalytically active phases (CAPs) in the reaction of ozone decomposition. All of them contribute to the total catalytic activity of SCWAs, but it is impossible to determine a specific contribution made by each of them.

The highest catalytic activity is demonstrated by SCWA-ANO, both FP and M, characterized by both high phase homogeneity and high CAP contents: 81 and 97 %, respectively. The low catalytic activity for FP SCWA-TsL can be explained not only by its low CAP content of 51 % but also by high contents of impurity phases such as calcium and nickel fluorides and magnesium silicate blocking an access to active sites of the SCWA surface for ozone molecules. Although the CAP contents are practically the same for FP and M SCWA-TsL (Table 4) and there is only a slight difference in the sizes of their Fe_3_O_4_ nanoparticles, removing water-soluble impurities makes accessible active phases of M SCWA-TsL taking part in the reaction of ozone decomposition and causes the steady-state mode of ozone decomposition (Fig. 3b, curve 1). In the case of FP SCWA-TsL and FP SCWA-UONI, their CAP contents are very close and a bigger activity of the latter in the reaction (Fig 3a) can be caused by other factors, e.g., by the great difference in the sizes of their ferrite nanoparticles. XRD (Fig. 1f) and IR spectroscopic analysis (Fig. 2f) show that substantial structural changes occurred after modification of SCWA-UONI. These changes resulted in a decrease of kinetic parameters (*W*_in_ and *k*_1_) (Table 5) characterizing the activity of M SCWA-UONI over the initial period of the reaction.

## Conclusion

The samples of SCWAs were obtained as a result of steel welding by three electrodes of Ukrainian manufacture. The phase compositions of the samples, both freshly prepared (FP) and modified (M) by water treatment at 60 °C, were studied by X-ray phase analysis and IR spectroscopy. The results of X-ray phase analysis show that the freshly prepared and modified SCWAs are polyphase systems consisting of magnetite, metal-substituted ferrites, metal oxides, carbonates, and fluorides of alkali and alkaline-earth metals, and chromates. The unit cell parameter and the size of a magnetite nanoparticle (ranging from 15 to 68 nm depending on the chemical composition of an electrode) have been estimated. IR spectral investigations confirm the polyphase composition of the SCWAs. The biggest differences are apparent in the regions of deformation vibrations of M–O–H bonds and stretching vibrations of M–O bonds (M–Fe, Cr).The kinetics of ozone decomposition by FP and M SCWAs at ${C}_{{\mathrm{O}}_3}^{\mathrm{in}}=100\ \mathrm{mg}/{\mathrm{m}}^3$ has been studied, and kinetic parameters (*W*_in_, *k*_1_, and *k*_1/2_) as well as amounts of ozone entered into the reaction have been calculated. The catalytic activity has been found to decrease in the order SCWA-ANO > SCWA-UONI ≈ SCWA-TsL corresponding to the decrease in the content of catalytically active phases in their compositions. A low activity of SCWA-UONI and SCWA-TsL is caused by a high content (20–24 %) of the calcium fluoride phase blocking a surface of the catalytically active phases.
